# When Education Begins

**Published:** 1872-07

**Authors:** 


### WHEN EDUCATION BEGINS.

It should begin a year before the child is born, before marriage, and if twenty years earlier, so much the better; for then the chances would be greater that the youthful pair should meet at the marriage altar in physical health, vigorous and permanent, with all the bodily functions matured, regular, and perfect.

There should be no concealed malady, no burrowing disease, no ailments of even a month's or day's duration; should there be any sickness whatever, the marriage ceremony, or its consummation, should be deferred until the system has been restored to its natural healthy condition; because whatever may be its state, whether of body or brain, at the instant of efficient congress, that condition will be imposed on the child to be born therefrom.

Authentic cases are recorded where persons have been so enraged, that within half an hour or less, the eyes and face have been diffused with a yellow tinge, showing that the entire blood of the system has been transformed; and as out of this blood the materials for the new being are drawn, it is a physiological impossibility that an impress should not be made on the physical, and mental, and moral constitutions foreign to nature and to health; precisely, it has been ascertained, as a child begotten in a drunken stupor will be idiotic; or will be prone to brain diseases or actual insanity, if begotten when the parent was "excited with liquor," having "taken a glass with a friend," — a very little thing indeed, in the estimation of some, but capable, under the combinations stated, of laying the foundations for untold misery, the misery of a mad-house for a life-time, to a human being capable otherwise of the highest human achievements.

If a transient sensation of the parent may give to a child the impress of that parent's character, much more will feelings which have been indulged in for days and weeks and months together, be incorporated into the very being, physical, mental, and moral, of the child born after them.

If any intelligent pair, in any community, should become parents in the practical observance of the principles inculcated in these pages, their children would not die early, but would mature and fructify, and thus become living springs themselves, sending out healthful progenies far and near, until the whole land would be peopled with inhabitants healthy, happy, and good, because "Like produces like;" because the time is divinely decreed to come, when it shall no longer be said that "The fathers have eaten sour grapes, and the children's teeth are set on edge." That good time can never come until the fathers cease to eat sour grapes; until they cease to be vicious, until they cease to riot in animal appetites and passions for the enjoyment of them, but shall gratify them in temperance, as a means of fulfilling the eternal design of replenishing the earth with the highest type of manhood.

As an incentive to cultivate aspirations like these, there is the great truth that evil and disease are not eternal; they are destined to death; while goodness of heart and mental activity are immortal. The latter must "increase," while the former must "decrease." The tiny mustard-seed must grow and fructify, and give its cooling shade, indefinitely extending. The little "stone cut out of the mountain without hands," shall roll onward, increasing ever, until the world shall be brought beneath it. The cloud, no bigger than a man's hand, must cover the whole sky, and pour its living rain on all that grows. All evil is for a day; all good for unending ages. But while these considerations are written for our comfort and encouragement, it must not be forgotten that vice and disease are to be exterminated, are to be hunted from the world, by the aggressive action of good men and women, an action which begins at the root of the matter, an action which removes the cause; then the effect ceases by its own limitation, ceases in the very nature of things. It cannot be that evil will in the end triumph.

In plainer phrase, if the children to be begotten are born healthy in body, mind, and morals, their descendants, in turn, will be like them, while the evil race now living perishes from the world. Such results will not come of themselves; they must be wrought out like every other good; they come by planning beforehand, and then carrying the plans into execution.

There must be a beginning somewhere; that beginning must be made in two human hearts, elevated, cultivated, conscientious. And infinite blessings must come for time and for aye upon that man and woman who, for the glory of God and the good of the race, shall set out to do all they can to make a beginning, to sow the first seed which would bear a fruitage so helpful to the world of mankind.

